# PERCEIVE – Patterning Employment, Race, and Clinical Experience In Violence against Employees

**DOI:** 10.1192/bjo.2021.873

**Published:** 2021-06-18

**Authors:** Ben McClure, Keith Reid

**Affiliations:** 1 Hopewood Park Hospital, Cumbria, Northumberland Tyne Wear NHS Foundation Trust; 2 Cumbria, Northumberland Tyne and Wear Foundation Trust, Northumbria University

## Abstract

**Aims:**

PERCEIVE is a service evaluation counting whether nurse demographics correlate with reported subjection to violence and verbal aggression. The setting was a large mental health, learning disability and neuropsychiatry NHS trust in England. This continues our work to understand correlations reported in the literature between temporary staff and violence.

**Method:**

We consulted the Caldicott, legal, equality & diversity, teams and gained service evaluation permission SER-19-031 from CNTW R&D department. We briefly consulted with staff regarding themes relevant to temporary nurse workers. They expressed concern that staff perceived to be “other” would be at more risk.

Employees’ age, ethnicity, employment status, nationality, length of service and seniority are routinely collected for the running of the trust. Therefore, these were anonymously collated then cross-referenced with violence and aggression incident reports (VA IR1s). Chi-squared was used to identify statistical significance. Ethno-national status was taken from self-report. We could not control for hours worked nor could we get agency staff demographic data.

We compared “exposure to at least one violent incident” in June, July and August 2019 against the following demographic categories:

Substantive vs bank staff

Band 5 and above vs band 4 and below

Staff with < 1 year of service vs staff with ≥ 1 year of service

“White British” staff vs Non-“White British” staff

“British” staff on self-report vs “Non-British” staff

Age ≤30 years vs ≥ 31years

A minimum of 1682 nursing staff were analysed for each category in each month.

**Result:**

Substantive staff, “White British”, “British”, younger, and staff of shorter employment length had greater frequencies of at least one VA IR1s compared to the complementary groups. Length of service was significant only in two months but judged significant overall. There was no statistically significant correlation with seniority. Substantive staff have three times the risk vs bank staff, perhaps mediated by hours worked. Other risk ratios were in the region x1.2 to x1.8.

**Conclusion:**

Being British, White British, younger, less experienced or substantive staff correlate with subjection to reported aggression. This did not fit with staff speculation during consultation. Survival effects may be relevant. We are working to get more detailed information. Induction may help reduce aggression against newer staff.

